# Strain-Dependent Gene Expression during Mouse Embryonic Palate Development

**DOI:** 10.3390/jdb3010002

**Published:** 2015-02-06

**Authors:** Jiu-Zhen Jin, Jixiang Ding

**Affiliations:** 1 Department of Molecular, Cellular and Craniofacial Biology and Birth Defects Center, University of Louisville School of Dentistry, Louisville, KY 40202, USA; 2 Division of Cardiovascular Medicine, University of Louisville School of Medicine, Louisville, KY 40202, USA

**Keywords:** secondary palate, *Meox-2*, *Fgfr1*, Swiss Webster, Black Swiss and C57B6

## Abstract

The effect of strain background on gene function in growth and development has been well documented. However, it has not been extensively reported whether the strain background affects the gene expression pattern. Here, we found that the expression of homeobox gene *Meox-2* and FGF receptor 1 gene *Fgfr1* during mouse palate development is strain-dependent. On the C57B6 inbred background, *Meox-2* is expressed in the palatal outgrowth on Embryonic Day 11.5 (E11.5); the expression shifts posteriorly and is restricted to the back of palate on E14.5. On the Swiss Webster outbred background, *Meox-2* expression covers both anterior and posterior regions with the same intensity from E12.5 to E14.5. On the Black Swiss background, *Meox-2* expression also covers the entire palate A-P axis, but is much weaker in the anterior region on E14.5. *Fgfr1* also displays distinct expression patterns in the palatal outgrowth on E11.5 in these three strains. On the Black Swiss outbred background, the expression is restricted to the anterior palatal outgrowth. In marked contrast, the expression in the Swiss Webster outbred strain is located exclusively in the posterior palate outgrowth on E11.5, whereas in the C57B6 inbred strain, the expression is undetectable in the palatal outgrowth on E11.5.

## 1. Introduction

Disruption of secondary palate development will lead to cleft palate, a common birth defect that affects 1:700 births [1]. The formation of the continuous secondary palate is a complex developmental process involving a series of steps, such as palate specification and initiation, vertical palatal growth, elevation and fusion [2,3]. During mouse embryogenesis and organogenesis, neural crest-derived mesenchymal cells and cranial ectoderm-originated epithelial cells form a cluster of bilateral facial primordia, including the two maxillary processes [2,4]. On Embryonic Day 11.5 (E11.5), a group of cells within these two maxillary processes are specified to become palatal mesenchymal cells, protrude into the primitive oral-nasal cavity and form two palatal outgrowths there [5]. On E12.5, the palatal outgrowth further develops to form the characteristic palate shelf that contains a core mass of mesenchymal cells enclosed by epithelial cells [1,4,5].

Vertical growth of the palatal shelves along the lateral aspects of the tongue continues into E13.5. On E14.5, however, the vertically-orientated palatal shelves undergo re-orientation and position themselves horizontally above the tongue, a process termed palate re-orientation or elevation [1–3]. The two re-orientated palatal shelves grow horizontally towards each other to meet along the facial midline. This contact induces the merging of the medial edge epithelium (MEE) of the two shelves to form the medial edge seam (MES) that will subsequently undergo degeneration, leading to mesenchymal confluence and the formation of a continuous palate, a process called palate fusion. The resulting continuous palate separates the primitive oral-nasal cavity into nasal and oral cavities [1–3].

The aforementioned processes have to be precisely regulated at the gene function level to assure the proper formation of secondary palate. Gene targeting technology has greatly advanced our understanding of the gene functions in palate growth, re-orientation and fusion, since it allows investigators to create mutations virtually in any genes of interest to study their function during development *in vivo* [1,3,6]. However, the mutant phenotype is often influenced by strain background. The effect of strain background on gene targeting was first reported in 1995 on the EGF receptor gene (*Egfr*) knock-out [7,8]. Loss of *Egfr* function leads to peri-implantation lethality on a CF-1 background [8], death at mid-gestation stage on the 129 background [7,8], lethality at birth on C57B6 [7] and post-natal lethality around 20 days of birth on MF-1 and CD-1 strain backgrounds [7,8]. Further studies uncovered a strain-dependent neurodegeneration defect in the *Egfr* knock-out mice [9]. Since then, the effect of strain background on knock-out mice has been supported by numerous studies and has become the consensus of the field [10]. The effect of strain background on gene function during mouse secondary palate development has also been described recently [11]. The mechanisms underlying the strain effects are not yet well understood.

In contrast to gene function studies, little effort has been made to analyze the effects of strain background on gene expression during mouse embryogenesis, either in secondary palate formation or in embryonic development in general. We reasoned the possibility that different strains may bear sequence variation in a gene regulatory region, which could, in principle, affect its expression, and decided to test this idea by searching for genes that give distinct expression patterns on different strain backgrounds. As a result of this effort, we reported in this study that *Meox-2* and *Fgfr1* displayed different expression patterns in mouse secondary palate development in C57B6, Black Swiss and Swiss Webster, three strains commonly used in mouse developmental biology studies. This is the first study reporting a given gene that displays distinct expression patterns, not levels, on different strain background during embryonic development.

## 2. Materials and Methods

### 2.1. Mice and Embryos

The Swiss Webster is an outbred line originated from Swiss mice, and Black Swiss is an outbred line generated by crossing N:NIH Swiss outbred mice with C57BL/6N, followed by a series of selections (http://www.criver.com). In contrast, C57B6 is an inbred line developed from mating of female 57 with male 52 (http://jaxmice.jax.org).

In this study, C57B6, Black Swiss and Swiss Webster mice were purchased from Taconic, USA (http://www.taconic.com). To collect embryos at various stages, timed matings were set up between male and female mice, and the day a vaginal plug observed was designated as Embryonic Day 0.5 (E0.5). In this study, embryonic heads from E11.5 to E14.5 were collected and fixed in 4% paraformaldehyde overnight followed by three time washes in PBS with 0.1% Tween-20 (PBT), 5 min each. The embryonic tissues then underwent dehydration through 25%, 50% and 75% methanol in PBT and were finally stored in 100% methanol in −20 °C up to 3 months for whole mount *in situ* hybridization.

### 2.2. Plasmids, Probes and In Situ Hybridization

To detect the expression of *Meox-2* mRNA and *Fgfr1* mRNA, we generated cRNA probes from the plasmids that contained full length cDNAs of *Meox-2* and *Fgfr1*. The plasmid for *Meox-2* has been described previously [12–14], and the plasmid for *Fgfr1* was from Dr. Janet Rossant’s laboratory [15]. Whole mount *in situ* hybridization was carried out according to the protocol described by Shen [16]. Briefly, digoxigenin-labeled antisense RNA probes were hybridized, followed by incubation with anti-digoxigenin-AP Fab fragments (Roche), which can be detected by a color reaction using NBT/BCIP (Roche). For a given stage, at least 10 embryos of each strain were examined.

### 2.3. Embryo Staging

As mentioned above, the day a vaginal plug observed was designated as Embryonic Day 0.5 (E0.5); however, it is very common for mouse embryos that the embryos on the same embryonic day could be developmentally varied, especially if the embryos are on a different strain background. We therefore applied a Theiler staging system (TS) (www.emouseatlas.org) and other criteria, such as mandibular arch morphology, limb bud and palatal rugae, to normalize the embryos from all three different strains. In brief, E11.5 embryos corresponded to TS 19, at which stage, the second branchial arch has not completely fused with the mandibular arch. E12.5 embryos corresponded to TS 20, in which the second branchial arch is completely fused to the mandibular arch, and the hindlimb, however, is still a smooth tissue without defined digits. E13.5 embryos resembled TS 21, at which stage, the hindlimb forms individual digits as the interdigital web tissue starts to break down, and the palatal rugae become visible. E14.5 embryos were similar to TS 22, at which stage, the hindlimb shows well-separated digits and the palatal rugae become more prominent.

## 3. Results and Discussion

*Meox-1* and *Meox-2* are two related mouse homeobox genes first discovered to be expressed specifically in paraxial mesoderm, such as somites, during development [12]. Subsequent studies revealed *Meox-2* expression in mouse secondary palate mesenchymal cells, and the expression specifically marks the posterior soft palate region in the C57B6 strain [13,17]. Loss of *Meox-2* function in C57B6 mice leads to a posterior cleft palate in 10%–20% of mutant embryos due to a post-fusion defect [14,18,19]. The present study explores whether strain background can affect *Meox-2* expression during mouse secondary palate development. Indeed, *Meox-2* displays distinct expression patterns on different strain backgrounds.

**Figure 1:**
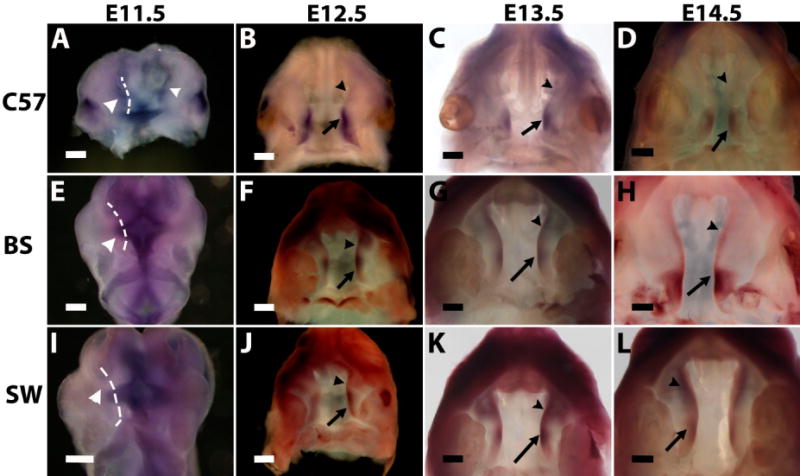
The expression of *Meox-2* during mouse secondary palate development on the backgrounds of C57B6 (**A**–**D**), Black Swiss (**E**–**H**) and Swiss Webster (**I**–**L**). Dashed lines in (**E**) and (**I**) indicate the unstained palate outgrowth areas. Arrows and arrowheads indicate to different regions in palate shelves, as illustrated in the Results and Discussion section. Scale bars represent 228 μm (**B**, **D**, **F**, **J**) and 285 μm (**A**, **C**, **E**, **G**–**I**, **K** and **L**); C57, BS and SW are the abbreviations for C57B6, Black Swiss and Swiss Webster.

As mentioned above, palatogenesis is initiated by the formation of an outgrowth out of the maxillary process on E11.5 [4,5]. On the C57B6 strain background, *Meox-2* expression can be detected as early as E11.5 in the palate outgrowth covering both anterior and posterior regions (Figure 1A). On E12.5, the expression of *Meox-2* is present only in the posterior two-thirds of the palate shelf (arrow in Figure 1B), but absent in the anterior region (arrowhead in Figure 1B). On E13.5, the expression further shifts to the posterior one-third of the palate shelf (arrow in Figure 1C) and is completely absent in the anterior two-thirds (arrowhead in Figure 1C). On E14.5, the expression is restricted to the posterior region corresponding to the soft palate (arrow in Figure 1D). No expression is found in the anterior hard palate (arrowhead in Figure 1D). This expression pattern of *Meox-2* confirmed the previous study using C57B [13,14].

On the Swiss Webster background, however, the expression of *Meox-2* is not detectable on E11.5 in the palate outgrowth (Figure 1I). On E12.5, unlike C57B6, *Meox-2* is expressed along the entire palate A-P axis, although the intensity is stronger in the posterior region (arrow in Figure 1J) than the anterior region (arrowhead in Figure 1J). From E13.5, the intensity of anterior expression (arrowhead in Figure 1K) is similar to that in the posterior expression (arrow in Figure 1K). On E14.5, *Meox-2* expression covers both anterior and posterior regions with the same intensity (arrowhead and arrow in Figure 1L).

On the Black Swiss background, *Meox-2* expression is not detectable in the palate outgrowth on E11.5 (Figure 1E). On E12.5 and E13.5, expression is found in both the anterior and posterior regions, but the posterior expression level (arrow in Figure 1F,G) is higher than the anterior expression (arrowhead in Figure 1F,G). On E14.5, the expression in the posterior soft palate is increased and expanded (arrow in Figure 1H), whereas the expression in the anterior hard palate is further reduced and narrowed, but not absent (arrowhead in Figure 1H).

Post-hybridization sectioning of these embryos confirmed the previous studies [13,14] showing that the expression is restricted to the mesenchymal cells [20].

Therefore, the expression of *Meox-2* in secondary palate development is strain dependent. It is expressed on E11.5 only in the C57B6 strain, but not in Black Swiss and Swiss Webster strains. From E12.5 to E14.5, the expression of *Meox-2* shows strong A-P polarity on the C57B6 background, since the expression is restricted to the posterior region and completely absent in the anterior region. The expression on the Swiss Webster background shows no A-P polarity on E14.5, although the expression on E12.5 and E13.5 is stronger in the posterior region; the expression is the same intensity from anterior to posterior on E14.5. The expression on the Black Swiss background on E14.5 is in an intermediate category between the C57B6 and Swiss Webster backgrounds; the posterior expression is strong in the posterior, whereas the anterior expression is weak and narrow, but not absent.

Since the *Meox-2* mutant line was studied only on the C57B6 background, the biological significance of this expression divergence among the three strains is not clear. However, all three strains express *Meox-2* in the posterior soft palate, and only Swiss Webster expresses the gene in both the anterior and posterior with the same intensity, suggesting that *Meox-2* is likely to be more important in the posterior. Consistent with this speculation, the cleft palates found in *Meox-2* mutant embryos on the C57B6 background are posterior clefts [14]. In addition, *Meox-2* has been shown to play important roles in muscle differentiation during mouse embryonic development [18,19], and soft palate is composed mainly of muscle [2].

The function of the FGF receptor 2 gene (*Fgfr2*) in mouse secondary palate development has been studied by both loss-of-function and gain-of-function approaches. [21,22]. However, the function of *Fgfr1* in secondary palate has not been reported, since the *Fgfr1* null mutant embryos are early lethal due to gastrulation defects [15]. Conditional deletion of the *Fgfr1* gene in mouse secondary palate has not yet been published. In the current study, we found that the *Fgfr1* gene shows divergent expression patterns among different strains during palate development, especially at early stages.

**Figure 2:**
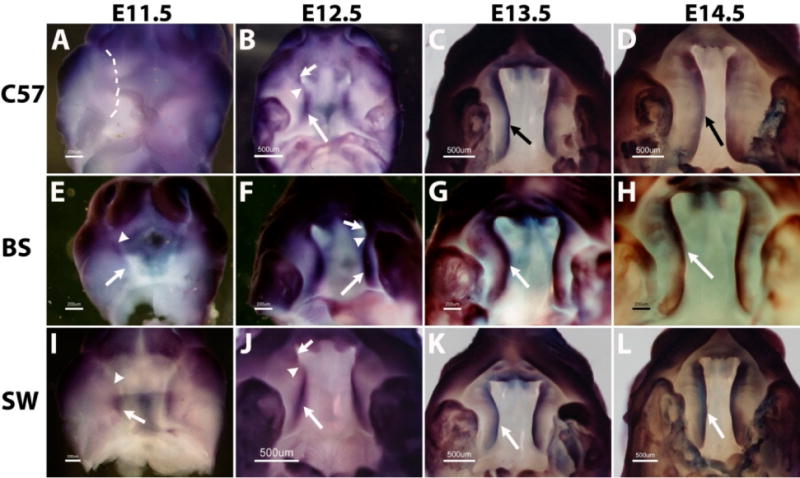
*Fgfr1* gene displays divergent expression patterns during mouse secondary palate formation among C57B6 (**A**–**D**), Black Swiss (**E**–**H**) and Swiss Webster (**I**–**L**) strains. The dashed line in (**A**) indicates the unstained palate outgrowth. Scale bars represent 200 μm (**A**, **E** and **I**) and 500 μm (**B**–**D**, **F**–**H** and **J**–**L**); arrows and arrowheads indicate different regions in palate shelves, as illustrated in the Results and Discussion section. C57, BS and SW are the abbreviations for C57B6, Black Swiss and Swiss Webster.

As shown in Figure 2, on the C57B6 background, *Fgfr1* expression is undetectable in the palate outgrowth on E11.5 (Figure 2A). On E12.5, high expression is found in the posterior region (arrow in Figure 2B) and the very anterior tip (short arrow in Figure 2B), but is absent in a portion of the anterior region (arrowhead in Figure 2B). From E13.5 to E14.5, the expression covers the entire A-P axis with the same intensity (Figure 2C,D).

Different from C57B6, the expression of *Fgfr1* in the Black Swiss strain is found in the palate outgrowth on E11.5 (Figure 2E), and the expression is restricted to the anterior half (arrowhead in Figure 2E). On E12.5, the expression covers the entire A-P axis of palate shelf with the same intensity in the anterior tip (short arrow in Figure 2F), posterior region (arrow in Figure 2F) and an anterior portion (arrowhead in Figure 2F). This uniform A-P expression pattern extends to E13.5 and E14.5 (Figure 2G,H).

In marked contrast to the Black Swiss strain, the expression of *Fgfr1* is only in the posterior region of the palate outgrowth on E11.5 in the Swiss Webster strain (arrow in Figure 2I). On E12.5, *Fgfr1* is highly expressed in the posterior region (arrow in Figure 2J), weakly expressed in the very anterior tip (short arrow in Figure 2J), but is almost absent in a portion of the anterior region (arrowhead in Figure 2J). From E13.5 to E14.5, similar to C57B6 and Black Swiss, *Fgfr1* expression on Swiss Webster is uniformly present along the entire palate A-P axis (Figure 2K,L).

Therefore, the expression pattern of *Fgfr1* in late palate development, E13.5 and E14.5, is similar among the three strains of C57B6, Black Swiss and Swiss Webster. However, the expression at early stages, especially the outgrowth stage on E11.5, shows considerable diversity in these three strains. Expression is undetectable on the C57B6 strain, in the anterior half only in the Black Swiss strain and the posterior half only in the Swiss Webster strain.

C57B6 and Black Swiss are two common inbred and outbred strains, respectively, used for gene targeting experiments. Swiss Webster is a common outbred strain widely used in gene expression studies. Despite the large number of studies regarding gene function on different strain backgrounds, very few studies explore the expression divergence of a given gene among different strains. It is likely that our findings with *Meox-2* and *Fgfr1* genes in secondary palate could also occur to other genes in other organs. Since the functions of *Meox-2* and *Fgfr1* in palate development have not been examined on different strains, the functional significance of this strain-dependent expression is not clear. However, the distinct expression of a given gene could, in principle, affect its function more or less and may account for the phenotype variations among different strains in gene targeting studies.

## 4. Conclusions

The data reported in this study demonstrate that gene expression patterns in the developing mouse secondary palate are affected by strain background. We have observed strain-dependent variation in gene expression specifically for the developing palate, but the phenomenon may occur in other organs, as well. These results emphasize that strain background must be considered when gene expression and gene function are analyzed and compared.
